# Experimental Colitis Is Attenuated by Cardioprotective Diet Supplementation That Reduces Oxidative Stress, Inflammation, and Mucosal Damage

**DOI:** 10.1155/2016/8473242

**Published:** 2016-01-06

**Authors:** Hilda Vargas Robles, Alí Francisco Citalán Madrid, Alexander García Ponce, Angelica Silva Olivares, Mineko Shibayama, Abigail Betanzos, Leonardo Del Valle Mondragón, Porfirio Nava, Michael Schnoor

**Affiliations:** ^1^Department of Molecular Biomedicine, CINVESTAV, Avenida IPN 2508, San Pedro Zacatenco, 07360 Mexico City, DF, Mexico; ^2^Department of Infectomics and Molecular Pathogenesis, CINVESTAV, Avenida IPN 2508, San Pedro Zacatenco, 07360 Mexico City, DF, Mexico; ^3^Department of Pharmacology, National Cardiology Institute “Ignacio Chávez”, 14080 Mexico City, DF, Mexico; ^4^Department of Physiology, Biophysics and Neurosciences, CINVESTAV, Avenida IPN 2508, San Pedro Zacatenco, 07360 Mexico City, DF, Mexico

## Abstract

Inflammatory bowel diseases (IBD) such as ulcerative colitis (UC) and Crohn's disease (CD) are multifactorial, relapsing disorders of the gastrointestinal tract. However, the etiology is still poorly understood but involves altered immune responses, epithelial dysfunction, environmental factors, and nutrition. Recently, we have shown that the diet supplement corabion has cardioprotective effects due to reduction of oxidative stress and inflammation. Since oxidative stress and inflammation are also prominent risk factors in IBD, we speculated that corabion also has beneficial effects on experimental colitis. Colitis was induced in male mice by administration of 3.5% (w/v) dextran sulfate sodium (DSS) in drinking water for a period of 3 or 7 days with or without daily gavage feeding of corabion consisting of vitamin C, vitamin E, L-arginine, and eicosapentaenoic and docosahexaenoic acid. We found that corabion administration attenuated DSS-induced colon shortening, tissue damage, and disease activity index during the onset of colitis. Mechanistically, these effects could be explained by reduced neutrophil recruitment, oxidative stress, production of proinflammatory cytokines, and internalization of the junctional proteins ZO-1 and E-cadherin leading to less edema formation. Thus, corabion may be a useful diet supplement for the management of chronic inflammatory intestinal disorders such as IBD.

## 1. Introduction

Inflammatory bowel disease (IBD) encompasses two idiopathic chronic inflammatory conditions: ulcerative colitis (UC) and Crohn's disease (CD) both of which are characterized by recurrent episodes of cramping, lower abdominal pain, diarrhea, bloody stools, weight loss, and chronic inflammatory changes of the mucosal tissue resulting in scarring and fibrosis [1]. The pathophysiology of IBD is still poorly understood but it clearly is multifactorial including genetic, nutritional, environmental, and immunological risk factors [2, 3]. Malnutrition is a common complication during acute relapses since nutrient absorption from the gastrointestinal tract is severely disturbed especially in CD [4]. Nutritional factors influence the initial vulnerability to the development of colitis [5] as well as the duration of disease remission [2, 5]. However, human studies investigating certain diets in IBD patients gave contradictive results. In general, high sugar consumption had detrimental effects while grain and vegetable consumption had rather beneficial effects [6, 7]. In animal models of experimental colitis, certain nutrient supplementations alleviated disease symptoms [8]. For example, L-arginine treatment ameliorated the disease activity index and epithelial permeability in mice treated with dextran sulfate sodium (DSS) due to reduced expression of proinflammatory cytokines and enhanced epithelial wound healing [9]. This effect was dependent on the generation of NO from L-arginine by iNOS because beneficial effects of L-arginine supplementation were absent in iNOS-deficient mice. Additionally, L-arginine applied directly into the colon was converted by the microflora into NO and reduced the inflammatory score but did not prevent tissue damage in DSS-treated mice [10]. DSS-treated rats receiving a mixture of amino acids consisting of methionine, threonine, and glutamate showed improved mucosal healing without changes of the inflammatory status [11]. Contradictive effects of antioxidative diet supplementations have been reported in colitis models. In the HLA-B27 transgenic rat IBD model, feeding of a mixture of glutathione, vitamin C, and vitamin E had no beneficial effect due to the fact that colitis progression was not paralleled by increased oxidative stress in this model [12]. By contrast, feeding DSS-treated mice with *γ*-tocopherol alleviated inflammation and mucosal damage during mild colitis but was ineffective at high DSS concentrations [13]. Also *ω*3-polyunsaturated fatty acids (*ω*3-PUFAs, such as eicosapentaenoic acid (EPA) and docosahexaenoic acid (DHA)) and *ω*3-PUFA-derived resolvins ameliorated symptoms of experimental colitis in mice and rats [14, 15]. However, little is known about effects of micronutrient mixtures combining antioxidative vitamins, anti-inflammatory PUFA, and an NO source. We recently showed that such a mixture has cardioprotective effects in obese mice by reducing blood pressure, triglyceride levels, production of reactive oxygen species (ROS), and proinflammatory cytokines [16]. Thus, we hypothesized that such a mixture of micronutrients will also have combinatorial beneficial effects on experimental colitis. Here, we show that a mixture consisting of the antioxidative vitamins C and E, the *ω*3-PUFAs eicosapentaenoic and docosahexaenoic acid, and L-arginine reduced disease activity index (DAI), oxidative stress, inflammation, and junction disassembly in colons of mice with DSS-induced experimental colitis. Thus, application of this mixture of micronutrients (corabion) as diet supplement may be helpful for the management of IBD.

## 2. Methods

### 2.1. Reagents and Antibodies

Dextran sulfate sodium (DSS; 40–50 kDa) was purchased from Affymetrix (Cleveland, OH). Occult bleeding was analyzed using the Hemoccult kit (Beckman Coulter, Mexico City, Mexico) according to the manufacturer's instructions. The following antibodies were used: anti-ZO-1, Alexa-labeled secondary antibodies (Thermo Fisher, Mexico City), and anti-E-cadherin (Santa Cruz Biotechnologies, Santa Cruz, CA).

### 2.2. Mice, DSS Colitis, and Corabion Treatment

C57Bl/6J male mice within a weight range of 21–25 g were used in colitis experiments. All experiments have been approved by the Institutional Animal Care and Use Committee of Cinvestav. In each experiment, all animals received food and water ad libitum over a period of 3 or 7 days, respectively. The colitis group received 3.5% DSS in drinking water. The colitis + corabion group was gavage-fed once daily a mixture of 200 mg/kg L-arginine, 83 mg/kg vitamin C, 46 mg/kg vitamin E, 77 mg/kg EPA, and 115 mg/kg DHA (corabion, kindly provided by Merck, Naucalpan, Mexico) in a 1 : 1 mixture of water and safflower oil. The control + corabion group received normal drinking water and the same daily dose of corabion. The disease activity index (DAI) consisting of weight loss, stool consistency, and peranal bleeding was determined daily. Values of 0–4 were given in a blinded fashion according to the severity of each parameter as described [17], so that the maximum DAI would be 12. After sacrificing the animals by cervical dislocation, colons were removed, their length recorded and then used in the experiments described below.

### 2.3. Histology

Paraffin cross sections of colon tissues on coated glass slides were stained with hematoxylin and eosin using standard protocols. Histological inflammation was assessed in a blinded fashion using a histological score for the degree of inflammation, mucosal inflammation, and crypt damage related to the percentage of the affected mucosal surface [17, 18].

### 2.4. Immunofluorescence Staining

Frozen tissue sections were mounted on glass coverslips, fixed in 100% ethanol for 20 min at −20°C, washed with PBS, and blocked for 1 h in 2% BSA and 10% normal goat serum. After overnight incubation at 4°C in primary antibodies, slides were washed and incubated for 1 h with species-specific fluorescently labeled secondary antibodies. Cover slips were mounted in Vecta-Shield medium (Vectorlabs, Burlingam, CA) and analyzed on a confocal laser microscope (FV-300, Olympus, Miami, FL).

### 2.5. Measurement of Biopterins

Reduced (tetrahydrobiopterin, BH4) and oxidized (dihydrobiopterin, BH2) forms of biopterins in mouse plasma were determined by capillary zone electrophoresis as described [19]. Plasma was deproteinized and filtered, before measurement using a P/ACETM MDQ electrophoresis system (Beckman Coulter, Mexico City, Mexico). A standard curve of biopterin dissolved in 6 mM phosphate buffer (pH 7.4) was used to calculate concentrations.

### 2.6. Fluorescence Microscopy of Oxidative Stress

Production of superoxides in the colons was visualized using the fluorescence dye dihydroethidium (DHE, Life Technologies, Grand Island, NY) as previously described [20]. Tissue cross sections on glass slides were incubated with 5 *μ*M DHE in water at 37°C for 30 min in the dark. Fluorescence of oxidized ethidium was recorded on a laser scanning confocal microscope (FV-300, Olympus).

### 2.7. RT-PCR

Total RNA from colon tissue samples was isolated using TRIzol (Invitrogen, Carlsbad, CA). Superscript II and oligo-(dT_12–18_) primers (Invitrogen) were used to synthesize cDNA. PCRs using Taq-DNA polymerase (Roche, Indianapolis, IN) were run on a Veriti 96-well thermal cycler (Applied Biosystems, Mexico City, Mexico). To prevent amplification of genomic DNA, forward and reverse primers (Uniparts, Mexico City, Mexico) were located in different exons: IL6-FW: CCTTCCTACCCCAATTTCCAA and IL6-RE: AGATGAATTGGATGGTCTTGGTC; IL1*β*-FW: GCAACTGTTCCTGAACTCAACT and IL1*β*-RE: TCTTTTGGGGTCCGTCAACT; Actin-FW: TATCCACCTTCCAGCAGATGT; Actin-RE: AGCTCAGTAACAGTCCGCCTA; IFN-FW: TCAAGTGGCATAGATGTGGAA; IFN-RE: TGGCTCTGCAGGATTTTCATG; KC-FW: TGTCAGTGCCTGCAGACCAT and KC-RE: CCTGAGGGCAACACCTTCA. PCR conditions were: 95°C for 5 min followed by 30 cycles of 95°C for 15 s, 58°C for 30 s, and 72°C for 30 s, plus a final extension for 10 min at 72°C.

### 2.8. Myeloperoxidase (MPO) Assay

Colon tissue was minced on ice in a beaker containing 1 mL of HTAB buffer and homogenized using a Polytron homogenizer. The homogenate was rinsed twice with 1 mL of HTAB buffer and sonicated again for 10 s, freeze-thawed three times, centrifuged at 14,000 rpm for 15 min, and assayed spectrophotometrically for MPO activity. Absorbance at 460 nm was measured using a Beckman DU-2 spectrophotometer (Beckman Instruments, Inc., Cedar Grove, NJ).

### 2.9. Statistics

All data are expressed as means ± standard deviation. Significance was evaluated using either Student's *t*-test or ANOVA. Statistical significance is expressed by *p* values less than 0.05.

## 3. Results

### 3.1. Mice Fed a Diet Supplemented with Corabion Develop Less Pronounced DSS Colitis

**Figure 1:**
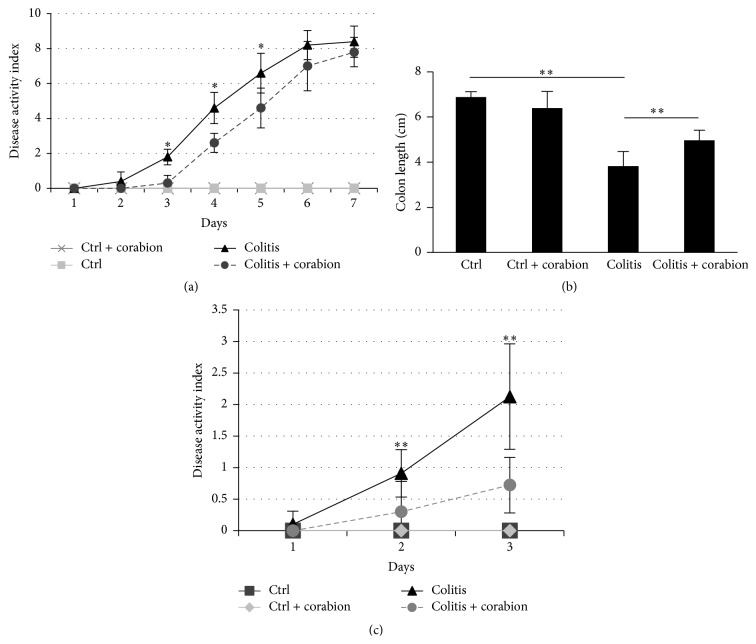
Corabion ameliorates disease activity index and colon shortening. (a) Disease activity index (DAI, values within 0–12 as described in Section 2) consisting of the parameters weight loss, stool consistency, and peranal bleeding of the four experimental groups: control (ctrl), colitis, colitis + corabion, and control + corabion. Control and control + corabion groups showed a DAI of 0 throughout the experimental period of seven days. *n* = 5 per group; ^*∗*^ *p* < 0.05. (b) Measurement of colon lengths at day 7. *n* = 5 per group; ^*∗∗*^ *p* < 0.01. (c) DAI of experimental groups for three days. These mice were sacrificed after three days and used for all further experiments. *n* = 12 per group; ^*∗∗*^ *p* < 0.01.

Corabion is a diet supplement consisting of vitamin C, vitamin E, L-arginine, and the *ω*3-PUFAs EPA and DHA. These substances have been applied alone with varying success in animal models of inflammation [2, 5, 8]. Since malnutrition and oxidative stress can trigger inflammatory disorders of the colon [2], we speculated that a combination of these micronutrients would have anti-inflammatory effects in vivo. Thus, we analyzed the effects of corabion on DSS-induced colitis (Figure 1(a)). Analysis of the DAI revealed that corabion treatment alone did not affect any of the parameters measured resulting in a DAI of 0 during the entire experimental period of seven days. By contrast, DSS treatment led to a continuously increasing DAI as expected reaching a maximum of 8.4 ± 0.89 on day seven. Corabion delayed the onset of colitis. The DAI was significantly lower on days 3, 4, and 5; but at days 6 and 7 corabion was no longer able to significantly ameliorate colitis. However, a significant reduction in colon length shortening was detected in the DSS + corabion group when compared with DSS + vehicle (Figure 1(b)). However, since the DAI was no longer significantly different at day 7, we decided to investigate the beneficial effects of corabion on DSS-induced colitis on day 3.  Figure 1(c) shows the DAI of independent groups consisting of 12 animals each. With this higher number of animals, even the changes with corabion on day 2 were statistically significant but the difference was more pronounced on day 3 (2.125 ± 0.83 for the DSS group versus 0.72 ± 0.44 for the DSS + corabion group). Thus, mice were sacrificed on day 3 and used for further experiments to investigate the nature of the observed protective effects.

### 3.2. Corabion Attenuates Colon Tissue Damage

**Figure 2:**
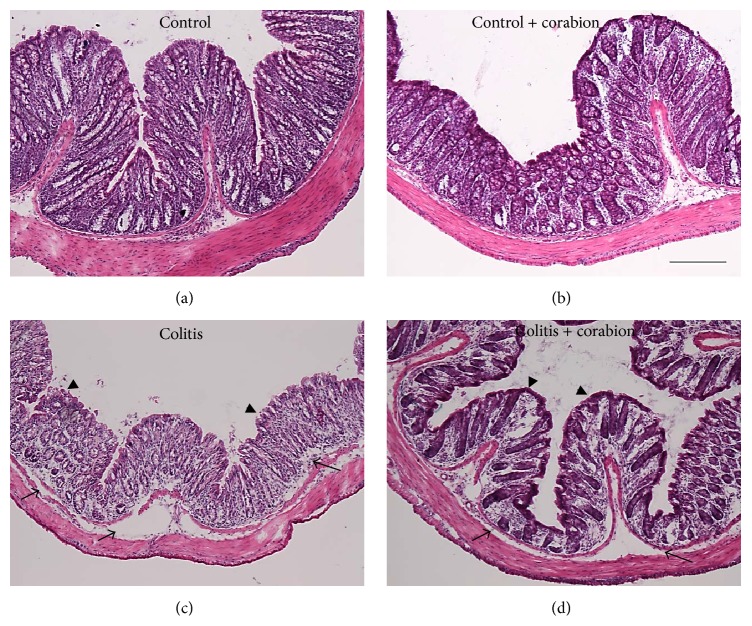
Colitis-induced tissue damage is less severe with corabion feeding. Paraffin cross sections were analyzed by hematoxylin/eosin staining: (a) control, (b) control + corabion, (c) colitis, and (d) colitis + corabion. Representative images of 3 independent tissue preparations per group are shown. Arrows indicate areas of edema and arrowheads indicate areas of apical erosions in the colitis group that are less pronounced in the colitis + corabion group. Images were taken with a 10x objective. Bar = 100 *μ*m.

We analyzed tissue morphology by hematoxylin/eosin (H/E) staining of paraffin embedded colon cross sections. Control samples showed the expected morphology (Figure 2(a)). Control animals treated with corabion only showed no significant differences in overall mucosal appearance but a tendency towards a denser apical epithelial surface (Figure 2(b)). Treatment with 3.5% DSS led to the typical signs of colitis, that is, edema formation, leukocyte recruitment, crypt shortening, and apical epithelial erosion (Figure 2(c)). Interestingly, parallel treatment with corabion clearly ameliorated the morphological changes observed with DSS treatment alone (Figure 2(d)). Edema formation, epithelial apical erosion, leukocyte infiltration, and crypt shortening were reduced and the overall morphology resembled the control more than the colitis group. Histopathological scoring of inflammation revealed significantly less diseases progression with parallel corabion treatment compared to the DSS alone group (Table 1). Although the total histopathological score was reduced by 40%, there was no significant difference in crypt damage and the changes were mostly attributed to inflammation and transmural extent (Table 1), very similar to what has been observed before with probiotic treatment [17]. Corabion alone did not significantly change the score compared to the untreated control group.

### 3.3. Inflammatory Neutrophil Recruitment and Oxidative Stress Are Reduced by Corabion

**Figure 3:**
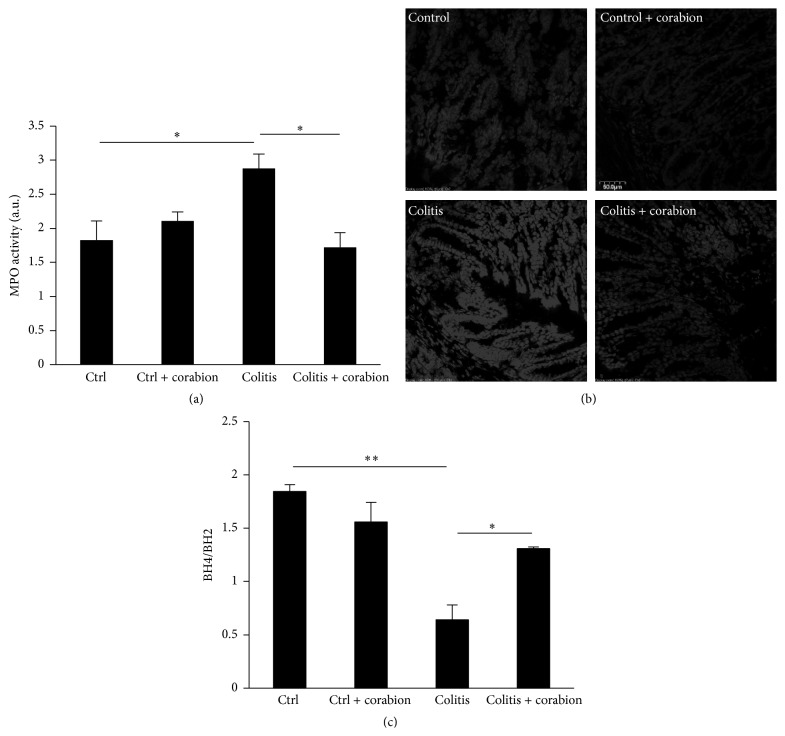
Corabion prevents neutrophil recruitment and oxidative stress. (a) Measurement of myeloperoxidase (MPO) activity as means of leukocyte recruitment in the four experimental groups: control (ctrl), colitis, colitis and corabion, and control and corabion. *n* = 4 per group; ^*∗*^ *p* < 0.05. (b) Fluorescence images of oxidized ethidium depicted in grayscales as means of ROS production and oxidative stress in colon tissues of the four experimental groups. Representative images of 3 independent tissue preparations are shown. Bar = 50 *μ*m. (c) Ratio of reduced tetrahydrobiopterin (BH4) to oxidized dihydrobiopterin (BH2) in mouse serum as measure of systemic oxidative stress is shown. *n* = 3; ^*∗*^ *p* < 0.05; ^*∗∗*^ *p* < 0.01.

Chronic inflammation in the colon is characterized by excessive neutrophil infiltration that contributes to tissue damage by releasing proteases and reactive oxygen species (ROS). Our histological analysis suggested that corabion prevented leukocyte recruitment during DSS-induced colitis. To corroborate this finding, we examined neutrophil recruitment in the colons of the experimental groups by means of MPO activity. As expected, we found robust neutrophil presence in response to DSS (Figure 3(a)). Importantly, corabion completely prevented DSS-induced neutrophil recruitment.

Since oxidative stress is another important feature of colitis, partly as a consequence of excessive neutrophil recruitment, we analyzed production of ROS in colon cross sections.  Figure 3(b) shows strongly increased oxidative stress in response to DSS. Already in basal conditions, corabion slightly decreased ROS production. Importantly, corabion treatment completely prevented the DSS-induced increase in oxidative stress (Figure 3(b)). Furthermore, we measured the ratio of reduced tetrahydrobiopterin (BH4) to its oxidized form (BH2) as marker of systemic oxidative stress in the circulation. This ratio was strongly reduced in response to DSS treatment (Figure 3(c)). Corabion treatment alone showed a tendency towards a decreased ratio but this did not reach statistical significance. By contrast, the reduction observed after DSS treatment was attenuated by corabion (Figure 3(c)) but not completely reversed as it was the case for ROS production.

### 3.4. Corabion Contributes to the Maintenance of TJ and AJ Architecture during Colitis

**Figure 4:**
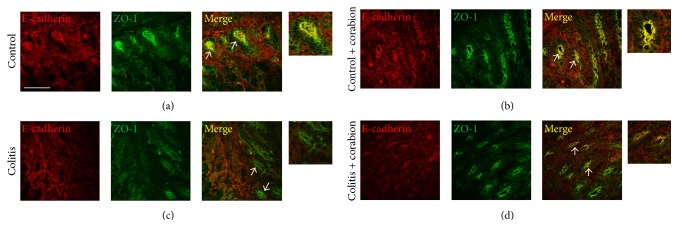
Junctional architecture is better preserved with corabion during colitis. Colon cryotissue sections were analyzed by immunofluorescence using antibodies against E-cadherin (red) and ZO-1 (green): (a) control, (b) ctrl + corabion, (c) colitis, and (d) colitis + corabion. Colocalization (yellow) of E-cadherin and ZO-1 at crypt epithelial cell contacts (arrows) is mostly lost in colitis (c) and much better conserved in colitis + corabion (d). A threefold digital zoom of selected crypts is shown to the right which better visualize the preserved colocalization with parallel corabion treatment during colitis (d). Representative images of 3 independent tissue preparations are shown. Bar = 50 *μ*m.

A critical factor contributing to colitis disease progression is epithelial permeability that is controlled in large part by the stability of intercellular junctions. Proinflammatory conditions contribute to the internalization of junctional proteins and destabilize epithelial cell contacts [21]. Since the overall tissue morphology was improved by corabion during DSS-induced colitis, we speculated that corabion could also protect tight and adherence junction molecules from being internalized during DSS-induced colitis. To this end, we performed immunofluorescence stainings of colon cryosections using antibodies against the TJ molecule ZO-1 and the AJ molecule E-cadherin. As shown in Figure 4, DSS induced a pronounced loss of E-cadherin and ZO-1 from epithelial cell contacts (Figure 4(c)), as compared to the control group where colocalization of E-cadherin and ZO-1 can be observed at crypt epithelial cell contacts (Figure 4(a)). Importantly, loss of E-cadherin and ZO-1 from crypt epithelial cell contacts was reduced when colitic mice were cotreated with corabion (Figure 4(d)). Moreover, the overall crypt structure looked less altered and was more comparable to the control stainings (Figures 4(a) and 4(d)). Corabion treatment alone preserved crypt morphology and junction composition (Figure 4(b)).

### 3.5. Production of Proinflammatory Cytokines in the Colon Is Reduced by Corabion

**Figure 5:**
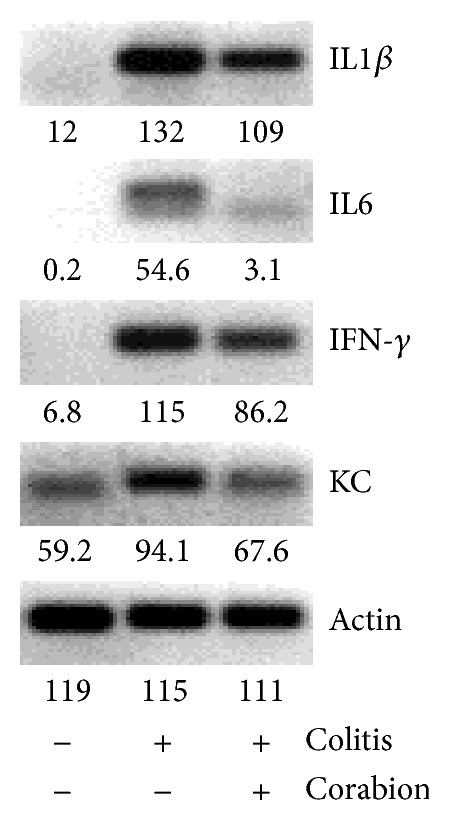
Corabion diminishes the production of certain proinflammatory cytokines. RNA was isolated and cDNA was synthesized from colons of the mice of the experimental groups: control, colitis, and colitis + corabion. Production of IL1*β*, IL6, IFN-*γ*, and KC mRNAs as measure of an early inflammatory response was determined by RT-PCR. *β*-actin was used as housekeeping gene. Pixel intensities as measured using Image J are indicated below each band. Representative images of triplicates from three animals per group are shown.

During colitis, proinflammatory cytokines and chemokines are produced which trigger the immune response. Thus, we analyzed by RT-PCR if corabion affected expression of such molecules. DSS treatment increased expression of IL1*β*, IL6, IFN-*γ*, and the most important chemoattractant for neutrophils in mice, keratinocyte-derived chemokine (KC), as expected (Figure 5) [22]. Importantly, while corabion was able to prevent DSS-induced increased expression of KC and IL6, IL1*β* and IFN-*γ* production was diminished (Figure 5).

## 4. Discussion

Here we show that a diet supplement developed to protect against cardiovascular disease (corabion) consisting of the antioxidative vitamins C and E, the NO source L-arginine, and the *ω*3-PUFAs EPA and DHA has also strong anti-inflammatory properties in a colitis mouse model. Corabion ameliorated DSS-induced tissue damage, leukocyte infiltration, oxidative stress, junction disassembly, and production of proinflammatory cytokines. Thus, corabion may be applied to prevent or at least attenuate IBD relapses.

Even though there is some evidence that nutrition can affect severity and relapse periods, there is still a lack of understanding of how nutritional supplements affect intestinal epithelial barrier functions during inflammation [6, 8, 23, 24]. A critical first line of defense against bacterial invasion and uncontrolled intestinal inflammation is a functional epithelial layer [3, 25, 26]. The epithelial monolayer is sealed by an array of intra- and intercellular protein-protein interactions, which form intercellular junctions consisting of the apically localized tight junctions (TJs) along with the adjacent adherens junctions (AJ) and desmosomes [27]. The stability of these integral transmembrane structures is affected by various agents such as commensal bacteria [17], immune cells [26], cytokines [28], and hormones [29]. It is commonly accepted that increased intestinal epithelial permeability due to junction disruption triggers intestinal inflammation [30]. However, it is still not well known how nutritional supplements affect IBD pathogenesis in general and epithelial barrier integrity in particular [7].

The DSS colitis model has been useful in such studies since it resembles several clinical signs of human IBD such as weight loss, diarrhea, inflammation, and epithelial barrier dysfunction [31]. Diet supplements have been tested in many studies to correlate their consumption to the development of experimental colitis with varying results [7, 8]. For example, *ω*3-PUFAs such as EPA and DHA have been shown to exert anti-inflammatory effects during DSS colitis in mice by reducing proinflammatory cytokine production and neutrophil infiltration leading to improved DAI and mucosal tissue damage [14]. DSS-induced colitis in rats was also ameliorated by *ω*3-PUFAs by improving tissue damage despite increased neutrophil infiltration [15]. However, no significant evidence was found in human studies that *ω*3-PUFAs alone would be sufficient to maintain remission in IBD patients [32]. These data show that even though beneficial effects are observed in animal models, *ω*3-PUFAs treatment has no significant effects in IBD patients when applied alone thus highlighting the necessity for combined diet supplements.

The amino acid L-arginine can regulate many signaling pathways because it is a substrate of NO synthase and thus a source of NO that affects IBD on various levels [4, 33]. While effects of NO in IBD are controversial and may highly depend on concentration and localized production [34], a more recent study showed beneficial effects of L-arginine supplementation in a DSS colitis mouse model [9]. Oral L-arginine application improved the disease activity index and mucosal tissue architecture, reduced leukocyte infiltration, and prevented proinflammatory cytokine production. Importantly, these effects could be attributed to NO production from L-arginine since the observed beneficial effects of L-arginine supplementation were lost when colitis was induced in iNOS-deficient mice [9]. On the other hand, pharmacological inhibition of iNOS alone did not affect the development of TNBS colitis in rats despite reduced NO production [35]; and high L-arginine doses aggravated colitis in TNBS-treated rats [36]. The microflora in the gut lumen can also produce NO [37]. A recent study investigated the effect of luminal NO production on the development of DSS colitis in mice by rectal administration of an NO donor [10]. Luminal NO production reduced leukocyte infiltration and epithelial permeability but did not prevent mucosal tissue damage during DSS colitis suggesting that bacterial NO production contributes to disease regulation. These data show that L-arginine due to NO production has certain beneficial effects but cannot account for the improvement of all different clinical signs of experimental colitis. Thus, it has been suggested that L-arginine may unveil its full protective potential only if combined with other micronutrients [4]. Clearly, more studies are required to unravel the localized and dose-dependent mechanisms of NO actions.

Oxidative stress, caused in part by excessive neutrophil infiltration, is another important characteristic of IBD [38]. Thus, antioxidants have also been studied in animal colitis models and IBD patients for their usefulness to ameliorate the disease. In particular, vitamin E and its derivatives, the tocopherols, have been extensively studied and are known for their antioxidative and anti-inflammatory properties [39]. *γ*-Tocopherol alone has been shown to ameliorate mild colitis induced by 1.5% DSS [13]. However, in the same study, a mixture of tocopherols including *γ*-tocopherol was not effective in the prevention of mild colitis. Colon carcinogenesis induced by combined DSS and azoxymethane was also attenuated by *γ*-tocopherol but not by a tocopherol mixture [13]. However, another study reported that a tocopherol mixture was effective in reducing inflammation and carcinogenesis in DSS and azoxymethane-treated mice. The discrepancies are likely to be explained by the different mouse strains and different concentrations of tocopherols used in their mixtures but they show that variances in the experimental setting can greatly affect the outcome of a study. Moreover, in HLA-B27 rats, diet supplementation with vitamins C and E and glutathione had no effect on colitis development [12]. However, oxidative stress in the colon did not increase much in this colitis model explaining the ineffectiveness of antioxidants in this study. By contrast, vitamin E protected rats against TNBS-induced colitis by reducing tissue damage, lipid peroxidation, and proinflammatory cytokine production [40]. However, neutrophil recruitment was not affected by vitamin E in this setting. Another study reported that vitamin E reduced oxidative stress in TNBS-treated rats without affecting tissue damage [41]. However, combination of vitamin E with selenium reduced both oxidative stress and tissue damage in this model. These data show that antioxidative micronutrients can have beneficial effects depending on the model system but that oxidative stress does not seem to be the major cause of inflammation and tissue damage since other micronutrients showed stronger beneficial effects on both inflammatory score and mucosal damage.

The discussed studies clearly show the necessity of defined studies using combinations of micronutrients that have the potential of ameliorating not only one (as expected from most single micronutrients) but various classical IBD features such as oxidative stress, production of proinflammatory cytokines, neutrophil infiltration, and epithelial dysfunction. We show here that the mixture of micronutrients used in corabion effectively counteracts the increases of these features observed during the well-defined DSS-induced colitis in C57Bl/6J mice. Given the mostly beneficial effects of the single corabion components, it was to be expected that the mixture would indeed combine all their beneficial effects. However, experimental evidence proving this assumption was lacking until now.

In summary, given its promising protective effects in the DSS colitis mouse model, corabion may serve as adequate diet supplement to reduce oxidative stress, inflammation, and tissue damage also in IBD patients.

## Figures and Tables

**Table 1:** Histopathological scores. H/E stainings of 3 independent tissue preparations of the experimental groups: control (ctrl), colitis (DSS), DSS + corabion, and ctrl + corabion were scored. Scoring of inflammation was done as described [17, 18].

	Ctrl	Ctrl + corabion	DSS	DSS + corabion	*p* value DSS versus DSS + corabion
Inflammation	0.17 ± 0.25	0.11 ± 0.22	3.56 ± 0.88	1.94 ± 0.72	0.002
Extent	0.28 ± 0.36	0.22 ± 0.36	4.03 ± 1.36	2.08 ± 0.88	0.003
Crypt damage	0.03 ± 0.08	0.06 ± 0.11	1.89 ± 0.78	1.72 ± 1.12	0.719
Total score	**0.48 ± 0.23**	**0.39 ± 0.23**	**9.48 ± 1.01**	**5.74 ± 0.89**	**0.016**

## References

[1] Xavier R. J., Podolsky D. K. (2007). Unravelling the pathogenesis of inflammatory bowel disease. *Nature*.

[2] Neuman M. G., Nanau R. M. (2012). Inflammatory bowel disease: role of diet, microbiota, life style. *Translational Research*.

[3] Colgan S. P., Curtis V. F., Lanis J. M., Glover L. E. (2015). Metabolic regulation of intestinal epithelial barrier during inflammation. *Tissue Barriers*.

[4] Cöeffier M., Marion-Letellier R., Déchelotte P. (2010). Potential for amino acids supplementation during inflammatory bowel diseases. *Inflammatory Bowel Diseases*.

[5] Shen W., Gaskins H. R., McIntosh M. K. (2014). Influence of dietary fat on intestinal microbes, inflammation, barrier function and metabolic outcomes. *Journal of Nutritional Biochemistry*.

[6] Yamamoto T. (2013). Nutrition and diet in inflammatory bowel disease. *Current Opinion in Gastroenterology*.

[7] Spooren C. E. G. M., Pierik M. J., Zeegers M. P., Feskens E. J. M., Masclee A. A. M., Jonkers D. M. A. E. (2013). Review article: the association of diet with onset and relapse in patients with inflammatory bowel disease. *Alimentary Pharmacology & Therapeutics*.

[8] Nanau R. M., Neuman M. G. (2012). Nutritional and probiotic supplementation in colitis models. *Digestive Diseases and Sciences*.

[9] Coburn L. A., Gong X., Singh K. (2012). L-arginine supplementation improves responses to injury and inflammation in dextran sulfate sodium colitis. *PLoS ONE*.

[10] Vermeiren J., Hindryckx P., Van Nieuwenhuyse G. (2012). Intrarectal nitric oxide administration prevents cellular infiltration but not colonic injury during dextran sodium sulfate colitis. *Digestive Diseases and Sciences*.

[11] Liu X., Beaumont M., Walker F. (2013). Beneficial effects of an amino acid mixture on colonic mucosal healing in rats. *Inflammatory Bowel Diseases*.

[12] Schepens M. A. A., Vink C., Schonewille A. J. (2011). Supplemental antioxidants do not ameliorate colitis development in HLA-B27 transgenic rats despite extremely low glutathione levels in colonic mucosa. *Inflammatory Bowel Diseases*.

[13] Jiang Q., Jiang Z., Hall Y. J. (2013). Gamma-tocopherol attenuates moderate but not severe colitis and suppresses moderate colitis-promoted colon tumorigenesis in mice. *Free Radical Biology & Medicine*.

[14] Bento A. F., Claudino R. F., Dutra R. C., Marcon R., Calixto J. B. (2011). Omega-3 fatty acid-derived mediators 17(R)-hydroxy docosahexaenoic acid, aspirin-triggered resolvin D1 and resolvin D2 prevent experimental colitis in mice. *The Journal of Immunology*.

[15] Varnalidis I., Ioannidis O., Karamanavi E. (2011). Omega 3 fatty acids supplementation has an ameliorative effect in experimental ulcerative colitis despite increased colonic neutrophil infiltration. *Revista española de enfermedades digestivas: organo oficial de la Sociedad Española de Patología Digestiva*.

[16] Vargas-Robles H., Rios A., Arellano-Mendoza M., Escalante B. A., Schnoor M. (2015). Antioxidative diet supplementation reverses high-fat diet-induced increases of cardiovascular risk factors in mice. *Oxidative Medicine and Cellular Longevity*.

[17] Mennigen R., Nolte K., Rijcken E. (2009). Probiotic mixture VSL#3 protects the epithelial barrier by maintaining tight junction protein expression and preventing apoptosis in a murine model of colitis. *The American Journal of Physiology—Gastrointestinal and Liver Physiology*.

[18] Dieleman L. A., Palmen M. J. H. J., Akol H. (1998). Chronic experimental colitis induced by dextran sulphate sodium (DSS) is characterized by Th1 and Th2 cytokines. *Clinical and Experimental Immunology*.

[19] Arellano-Mendoza M. G., Vargas-Robles H., Del Valle-Mondragon L., Rios A., Escalante B. (2011). Prevention of renal injury and endothelial dysfunction by chronic L-arginine and antioxidant treatment. *Renal Failure*.

[20] Mendoza M. G. A., Castillo-Henkel C., Medina-Santillan R. (2008). Kidney damage after renal ablation is worsened in endothelial nitric oxide synthase (−/−) mice and improved by combined administration of L-arginine and antioxidants. *Nephrology*.

[21] Bruewer M., Utech M., Ivanov A. I., Hopkins A. M., Parkos C. A., Nusrat A. (2005). Interferon-*γ* induces internalization of epithelial tight junction proteins via a macropinocytosis-like process. *The FASEB Journal*.

[22] Yan Y., Kolachala V., Dalmasso G. (2009). Temporal and spatial analysis of clinical and molecular parameters in dextran sodium sulfate induced colitis. *PLoS ONE*.

[23] Hou J. K., Lee D., Lewis J. (2014). Diet and inflammatory bowel disease: review of patient-targeted recommendations. *Clinical Gastroenterology and Hepatology*.

[24] Wu G. D., Bushmanc F. D., Lewis J. D. (2013). Diet, the human gut microbiota, and IBD. *Anaerobe*.

[25] Ivanov A. I. (2012). Structure and regulation of intestinal epithelial tight junctions: current concepts and unanswered questions. *Advances in Experimental Medicine and Biology*.

[26] Sumagin R., Parkos C. A. (2015). Epithelial adhesion molecules and the regulation of intestinal homeostasis during neutrophil transepithelial migration. *Tissue Barriers*.

[27] Turner J. R. (2009). Intestinal mucosal barrier function in health and disease. *Nature Reviews Immunology*.

[28] Ivanov A. I., Naydenov N. G. (2013). Dynamics and regulation of epithelial adherens junctions: recent discoveries and controversies. *International Review of Cell and Molecular Biology*.

[29] Hayashi Y., Narumi K., Tsuji S. (2011). Impact of adrenomedullin on dextran sulfate sodium-induced inflammatory colitis in mice: insights from in vitro and in vivo experimental studies. *International Journal of Colorectal Disease*.

[30] Schmitz H., Barmeyer C., Fromm M. (1999). Altered tight junction structure contributes to the impaired epithelial barrier function in ulcerative colitis. *Gastroenterology*.

[31] Perše M., Cerar A. (2012). Dextran sodium sulphate colitis mouse model: traps and tricks. *Journal of Biomedicine and Biotechnology*.

[32] Turner D., Shah P. S., Steinhart A. H., Zlotkin S., Griffiths A. M. (2011). Maintenance of remission in inflammatory bowel disease using omega-3 fatty acids (fish oil): a systematic review and meta-analyses. *Inflammatory Bowel Diseases*.

[33] Cross R. K., Wilson K. T. (2003). Nitric oxide in inflammatory bowel disease. *Inflammatory Bowel Diseases*.

[34] Kolios G., Valatas V., Ward S. G. (2004). Nitric oxide in inflammatory bowel disease: a universal messenger in an unsolved puzzle. *Immunology*.

[35] Armstrong A. M., Campbell G. R., Gannon C., Kirk S. J., Gardiner K. R. (2000). Oral administration of inducible nitric oxide synthase inhibitors reduces nitric oxide synthesis but has no effect on the severity of experimental colitis. *Scandinavian Journal of Gastroenterology*.

[36] Mañé J., Fernández-Bañares F., Ojanguren I. (2001). Effect of L-arginine on the course of experimental colitis. *Clinical Nutrition*.

[37] Sobko T., Reinders C. I., Jansson E. Å., Norin E., Midtvedt T., Lundberg J. O. (2005). Gastrointestinal bacteria generate nitric oxide from nitrate and nitrite. *Nitric Oxide*.

[38] Piechota-Polanczyk A., Fichna J. (2014). Review article: the role of oxidative stress in pathogenesis and treatment of inflammatory bowel diseases. *Naunyn-Schmiedeberg's Archives of Pharmacology*.

[39] Wallert M., Schmölz L., Galli F., Birringer M., Lorkowski S. (2014). Regulatory metabolites of vitamin E and their putative relevance for atherogenesis. *Redox Biology*.

[40] González R., Sánchez de Medina F., Gálvez J., Rodríguez-Cabezas M. E., Duarte J., Zarzuelo A. (2001). Dietary vitamin E supplementation protects the rat large intestine from experimental inflammation. *International Journal for Vitamin and Nutrition Research*.

[41] Ademoglu E., Erbil Y., Tam B. (2004). Do vitamin E and selenium have beneficial effects on trinitrobenzenesulfonic acid-induced experimental colitis. *Digestive Diseases and Sciences*.

